# Geriatric consultation services-are wards more effective than teams?

**DOI:** 10.1186/1741-7015-11-49

**Published:** 2013-02-22

**Authors:** Ian D Cameron, Susan Kurrle

**Affiliations:** 1 Rehabilitation Studies Unit, Sydney Medical School Northern, University of Sydney, Ryde, NSW, Australia; 2 Curran Chair in Healthcare of Older People, Sydney Medical School Northern, University of Sydney, Hornsby, NSW, Australia

**Keywords:** Acute care, functional status, geriatric consultation, multidisciplinary assessment

## Abstract

Geriatric consultation teams are one of the models for bringing comprehensive geriatric assessment to vulnerable and frail older people in the acute care hospital setting. While ward-based comprehensive geriatric assessment has been established as effective with reference to improving functional status and other outcomes, the team-based variant remains unproven for outcomes other than mortality in the medium term, as shown in a recent study published in *BMC Medicine *by Deschodt and colleagues. Further research might establish the effectiveness of the team-based model but, for current clinical practice, the emphasis should be on streaming older people with complex problems needing multidisciplinary assessment and treatment to ward-based models of comprehensive geriatric assessment.

## Background

Internationally, hospitals are experiencing an increasing demand for acute hospital services for older people. It is inevitable that this trend will continue with the aging of the population in most countries [1]. The prevalence of multimorbidity that is likely to be associated with hospitalization is substantial.

Comprehensive geriatric assessment (CGA), sometimes termed geriatric evaluation and management (GEM), is an established health technology. An accepted definition of CGA is "a multidimensional interdisciplinary diagnostic process focused on determining a frail older person's medical, psychological and functional capability in order to develop a coordinated and integrated plan for treatment and long term follow up" [2]. There are a number of published systematic reviews and meta-analyses that, generally, are strongly in support of this method of provision of care for vulnerable, frail older people in hospital care [3-5].

Within the scope of CGA, a number of models have been developed. Some of these are based on a discrete ward or geographically designated beds, others involve consultation teams. Ward-based services often involve direct care by the geriatric medicine team of the older patient, and some are shared care between admitting physician, or surgeon, and the geriatric medicine team. Some of these models occur within designated CGA wards, and some in general medical or surgical wards. To date, geographically-based wards/beds seem to be more effective at implementing CGA. This may be because the recommendations formulated as a result of the CGA are more likely to be implemented if there is a defined ward [6]. However, other explanations, such as staff expertise have been given [5].

## What are the benefits of CGA delivered by mobile teams?

One model of CGA is that which is delivered by a mobile team. In order to resolve whether or not CGA delivered by mobile inpatient geriatric consultation teams is, in fact, beneficial, Deschodt and colleagues performed a systematic review of the literature based on all relevant studies [6].

The authors found that geriatric consultation teams delivering CGA are not effective in terms of functional status, readmission or length of stay [4]. However, the research article found that there is probably a medium term reduction in mortality upon use of the geriatric consultation teams. Some individual trials within the systematic review reported clinically important effects.

This is a carefully conducted systematic review and meta-analysis. A strong case is made for the inclusion of non-randomized, but parallel control group, trials because of the practical difficulties of conducting these studies in the ward setting without contaminating the control group. Studies in acute medical and surgical ward settings have been included.

There appears to be improved medium term survival but not improvement in other outcomes, including function and discharge residence. What are we to make of this?

It seems that an improvement in functional status is not likely, based on the authors' careful treatment of this topic and the large amount of data that was included. Treatment effects are small and there is large heterogeneity. Perhaps this is not surprising when the studies were conducted in acute medical wards. In this setting, there is not a high priority placed on physical functioning, and the instruments used to measure function may have ceiling effects for the populations studied. Similarly, analyses of length of hospital stay, which were based on a smaller number of participants, seemed to be unchanged by CGA delivered by mobile teams.

The observed effects on mortality are hard to interpret. Why would there be an effect at the intermediate times (six and eight months) but not at one month and one year? The early estimate of mortality is based on only one study and the effect sizes up to eight months are roughly equivalent. We can accept that there is probably a medium term effect to reduce deaths. Equally, there was no effect on readmissions but high heterogeneity was noted.

Other, similar, systematic reviews have been conducted. For instance, one conducted by Ellis *et al. *[5], which is based on a Cochrane Collaboration Review that the authors have referenced, comes to similar conclusions. The seven randomized trials included in that review are also included in the current review. However, that review had some differences. It only included randomized trials, used "living at home" as its primary outcome, used fixed effects models if there was limited heterogeneity (I squared <= 30%), and did not have useable data for geriatric consultation teams for the outcome of "dependence". Use of a random effects model by Deschodt and colleagues is a more conservative approach which seems reasonable given the variety of studies considered.

The editorial accompanying the Ellis *et al*. systematic review, by Stuck and Iliffe, is strongly in favor of CGA [7]. Unfortunately, it does not address the important issue, which is highlighted by the current review, that team-based, rather than ward-based, CGA is not established as clearly effective when the CGA is provided on a consultation basis [6,5].

## Future directions

The major unanswered question is to what extent the findings of the Deschodt systematic review are due to limited efficacy of CGA as delivered by a team, or rather are due to inadequate adherence to the recommendations of the team. Investigation of adherence with recommendations should be undertaken. This review did not address the effect on function of modification of the ward environment, as described by Landefeld *et al*. in their study on acute care for older patients [8]. An older patient-friendly environment was felt to be important and may contribute to the effectiveness of ward-based CGA. It is also possible that uni-disciplinary interventions, particularly with reference to the nursing regime provided, might be effective and, therefore, should be investigated.

The cost effectiveness of CGA as delivered by a team is also an important area for further investigation. Very limited cost data are available for two of the studies in the current review studies but no conclusions can be drawn.

## Conclusions

The systematic review is highly relevant for decision makers struggling to address the needs of older, frail, acutely ill patients who have been admitted to the hospital and who are having their primary diagnosis addressed by an organ system-specific medical team, or surgical team, who have limited experience and expertise with these challenging and complex patients.

How should the results of this systematic review be used? Does this help discussion with the hospital administrator about how to improve treatment for vulnerable older people in the hospital?

As the authors noted, there is clear evidence of benefit for ward-based models of CGA, or perhaps better termed GEM. This should be the first item for discussion and these models should be developed if resources and local circumstances permit.

Overall, the systematic review suggests that caution should be applied to implementation of these consultation models due to the lack of evidence of efficacy. This is very reasonable given the demonstrated results. A crucial factor may be adherence to the recommendations of CGA, which has long been recognized as important [3].

A suitable approach might be to identify older people likely to benefit from CGA, for example, with advanced age (80 or 85 years), or with geriatric syndromes, and then stream these patients towards services with clear effectiveness (for example, ward-based services-GEM, stroke units or geriatric orthopedic programs). However, when talking with your hospital administrator, you cannot yet claim proven benefits of team-based CGA for your health service.

## Abbreviations

CGA: Comprehensive geriatric assessment; GEM: Geriatric evaluation and management

## Competing interests

The authors declare that they have no competing interests.

## Authors' contributions

IC and SK jointly drafted and wrote the commentary. Both authors read and approved the final manuscript.

## Pre-publication history

The pre-publication history for this paper can be accessed here:

http://www.biomedcentral.com/1741-7015/11/49/prepub
